# Criterion-Validity of Commercially Available Physical Activity Tracker to Estimate Step Count, Covered Distance and Energy Expenditure during Sports Conditions

**DOI:** 10.3389/fphys.2017.00725

**Published:** 2017-09-22

**Authors:** Yvonne Wahl, Peter Düking, Anna Droszez, Patrick Wahl, Joachim Mester

**Affiliations:** ^1^Institute of Biomechanics and Orthopedics, German Sport University Cologne Cologne, Germany; ^2^German Research Centre of Elite Sport, German Sport University Cologne Cologne, Germany; ^3^Integrative and Experimental Exercise Science, Department of Sport Science, University of Würzburg Würzburg, Germany; ^4^Department of Molecular and Cellular Sport Medicine, Institute of Cardiovascular Research and Sport Medicine, German Sport University Cologne Cologne, Germany

**Keywords:** wearables, validity, monitoring, biofeedback, athletes

## Abstract

**Background:** In the past years, there was an increasing development of physical activity tracker (Wearables). For recreational people, testing of these devices under walking or light jogging conditions might be sufficient. For (elite) athletes, however, scientific trustworthiness needs to be given for a broad spectrum of velocities or even fast changes in velocities reflecting the demands of the sport. Therefore, the aim was to evaluate the validity of eleven Wearables for monitoring step count, covered distance and energy expenditure (EE) under laboratory conditions with different constant and varying velocities.

**Methods:** Twenty healthy sport students (10 men, 10 women) performed a running protocol consisting of four 5 min stages of different constant velocities (4.3; 7.2; 10.1; 13.0 km·h^−1^), a 5 min period of intermittent velocity, and a 2.4 km outdoor run (10.1 km·h^−1^) while wearing eleven different Wearables (Bodymedia Sensewear, Beurer AS 80, Polar Loop, Garmin Vivofit, Garmin Vivosmart, Garmin Vivoactive, Garmin Forerunner 920XT, Fitbit Charge, Fitbit Charge HR, Xaomi MiBand, Withings Pulse O_x_). Step count, covered distance, and EE were evaluated by comparing each Wearable with a criterion method (Optogait system and manual counting for step count, treadmill for covered distance and indirect calorimetry for EE).

**Results:** All Wearables, except Bodymedia Sensewear, Polar Loop, and Beurer AS80, revealed good validity (small MAPE, good ICC) for all constant and varying velocities for monitoring step count. For covered distance, all Wearables showed a very low ICC (<0.1) and high MAPE (up to 50%), revealing no good validity. The measurement of EE was acceptable for the Garmin, Fitbit and Withings Wearables (small to moderate MAPE), while Bodymedia Sensewear, Polar Loop, and Beurer AS80 showed a high MAPE up to 56% for all test conditions.

**Conclusion:** In our study, most Wearables provide an acceptable level of validity for step counts at different constant and intermittent running velocities reflecting sports conditions. However, the covered distance, as well as the EE could not be assessed validly with the investigated Wearables. Consequently, covered distance and EE should not be monitored with the presented Wearables, in sport specific conditions.

## Introduction

In the past years, there was an increasing development of physical activity trackers (Wearables) which earned them the first place in the ACSM Worldwide Survey of Fitness Trends in 2016 and 2017, leaving popular topics like “High-intensity interval training” and “strength training” behind (Thompson, 2015, 2016).

Besides having applications for physical fitness and health in the general population by monitoring a plethora of different variables like step count, covered distance and energy expenditure (EE), Wearables may be useful for (elite) athletes as well. In these populations, Wearables might be used to monitor aspects of training load (Düking et al., 2016) as well as physical activity during leisure time and provide biofeedback to optimize exercises (Düking et al., 2017).

However, before Wearables can be used beneficially, the parameters they provide need to be scientifically trustworthy which implies that Wearables have sufficient validity which unfortunately is often an issue especially with commercially available Wearables (Sperlich and Holmberg, 2016). Several studies, recently summarized by Evenson et al. (2015) and Düking et al. (2016), tackled this issue and investigated the scientific trustworthiness of different Wearables under a variety of different conditions like walking, jogging, cycling, or resistance exercise under laboratory as well as under free-living conditions. Yet, scientific evaluations are strictly speaking only meaningful for the specific conditions the device was tested in and transfer of the results of these studies should be done carefully (Bassett et al., 2012). For recreational people, testing under walking or light jogging conditions might be sufficient. For (elite) athletes, however, scientific trustworthiness needs to be given for a broad spectrum of velocities or even fast changes in velocities reflecting the demands of the sport. There is scarce literature stating the validity of consumer level Wearables under sport specific conditions, even though some of the herein analyzed wearables are validated in the general population (El-Amrawy and Nounou, 2015; Alsubheen et al., 2016; An et al., 2017; Price et al., 2017).

Therefore the aim of the present study was to investigate the (concurrent) criterion-validity of eleven consumer Wearables concerning the amount of step count, covered distance and EE during running at four different velocities, an intermittent profile reflecting conditions in a soccer match and a 15-min outdoor trial at a constant velocity.

## Materials and methods

For the determination of the validity of step count, covered distance and EE, the criterion measures are described below. In order to test the validity of the eleven Wearables in a standardized situation under laboratory conditions, participants performed a running protocol of a total duration of 25 min, which consisted of four stages of different constant velocities lasting 5 min each, as well as a 5 min period of intermittent velocity. Validity for outdoor conditions was subsequently tested during a 15-min run at a constant velocity. The validity of the Wearables for step count, covered distance and EE was assessed during a single session of treadmill walking and running, using methods similar to previous validation studies (Takacs et al., 2014).

### Subjects and ethics statement

A total of 20 healthy and active sport students (10 male and 10 female) volunteered to participate in this study. All subjects gave written informed consent to the participation in the study. The study was performed in accordance with the declaration of Helsinki and approved by the Ethic Committee of the German Sport University Cologne.

### Instruments

#### Criterion measures

The Optogait system (OPTOGait, Microgate Srl, Bolzano, Italy) was used as the criterion measure for monitoring step count on the treadmill. The system is integrated within the sidebars of the treadmill (Pulsar, h/p/ cosmos sports and medical GmbH, Traunstein, Germany) and uses a photoelectric cell system to precisely measure the number of step count, which is a reliable (ICC = 0.962) and valid (ICC = 0.997) method for measuring step counts during treadmill trials (Lee et al., 2014). Step count was additionally assessed by a manual counter, which was also used in the outdoor condition.

The covered distance measured by the treadmill was used as a criterion measure and was determined based on the calibrated treadmill output (displayed on the electronic output of the treadmill in meters, based on the speed of the treadmill belt and time for each revolution of the belt) according to Takacs et al. (2014). The slope of the treadmill was automatically set at 1%.

The Metamax 3B (Metamax 3B, CORTEX Biophysik GmbH, Leipzig, Germany) is a portable gas analyzer allowing measurements of oxygen uptake under laboratory and free-living conditions, which was used in this study to calculate EE via indirect calorimetry as the criterion measure for EE. For the calculation of EE, oxygen uptake (VO_2_) was measured continuously breath by breath during the whole exercise and calculated according to previous reports (Scott et al., 2006). Before each session, the Metamax 3B flowmeter and gas analyzers were calibrated using a 3-liter syringe and a known gas mixture (15% O_2_ and 5% CO_2_). During calibration of the gas analyzer (O_2_ and CO_2_ sensors), the Metamax3B alternates sampling of the known gas mixture and ambient air. The Metamax 3B is a valid and reliable system for measuring oxygen uptake (Vogler et al., 2010). Methods of indirect calorimetry are the most commonly used to quantify human EE in both laboratory and field settings, typically by measuring oxygen uptake (Hills et al., 2014).

#### Wearables

Eleven Wearables were tested, including: Bodymedia Sensewear MF (300€, BodyMedia Inc, Pittsburgh, PA), Polar Loop (50€; Polar Electro, Kempele, Finnland), Beurer AS80 (30€; Beurer GmbH, Ulm, Germany), Fitbit Charge and Fitbit Charge HR (80€, 100€; Fitbit Inc, San Francisco, CA), Garmin Vivofit (90€), Garmin Vivosmart (100€), Garmin Vivoactive (250€), Garmin Forerunner 920XT (470€) (Garmin, Olathe, Kansas), Withings Pulse O_x_ (100€) (Withings SA, Issy-les-Moulineaux, France), Xiaomi MiBand (15€; Xiaomi Inc, Beijing, China). All devices use a triaxial accelerometer; Garmin Vivoactive and Garmin Forerunner 920XT also include a GPS sensor. The Fitbit Charge HR and all Garmin devices also use heart rate to calculate EE using photoplethysmography or chest belt sensors, respectively.

### Exercise study protocol

After arriving in the laboratory, anthropometric (weight, height, body fat) and personal data (date of birth, sex, handedness) of the participants were collected and transferred to all devices. Afterward, eleven Wearables were fixed at the wrist in a randomized order. The Bodymedia Sensewear armband and one Withings Pulse O_x_ device were placed on the backside of the upper arm and the hip, respectively. For the measurement of heart rate of the Garmin Wearables, the participants were fitted with a heart rate chestbelt.

**Figure 1:**
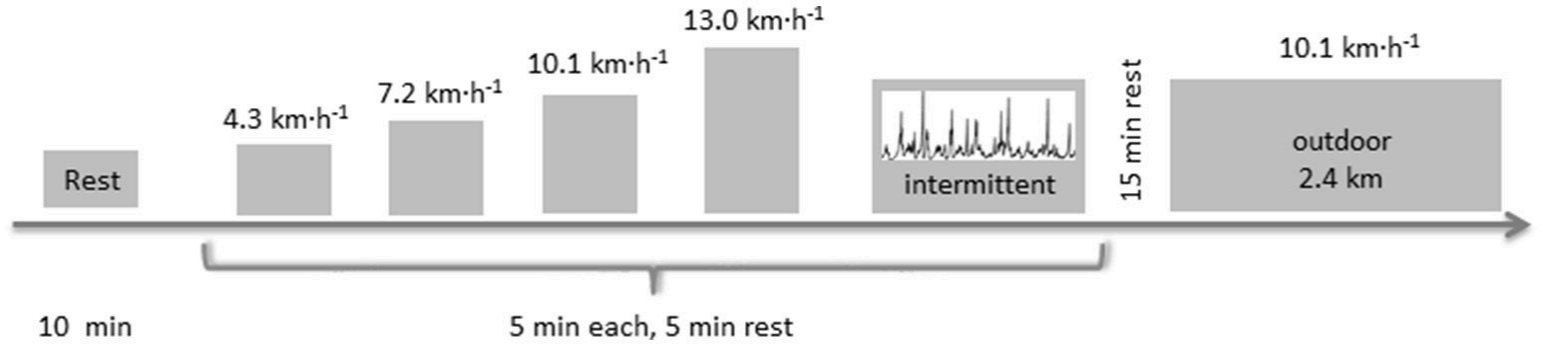
Exercise study protocol.

First, the participants were asked to lay down for 20 min. After the first 10 min, the measurement of resting EE was started using indirect calorimetry technique. Second, the running protocol was started, consisting of four 5 min stages of different constant velocities (walking: 4.3; 7.0; running: 10.1; 13.0 km·h^−1^) each separated by 5 min of passive rest. After these constant velocities stages, a 5 min period of intermittent velocity followed. This protocol was extracted from a smoothed running trial during a real soccer match (Amisco Data from a soccer match of the 1. German soccer league). The mean running velocity was 9.1 km·h^−1^, including twelve sprints with a maximal velocity of 22.4 km·h^−1^. Maximal acceleration and deceleration were 5.47 km·h^−2^ (1.52 m·s^−2^) and −4.88 km·h^−2^ (−1.36 m·s^−2^), respectively. Remaining time was covered with walking, defined by velocities smaller than 7.33 km·h^−1^, which is considered as preferred transition speed between walking and running (Rotstein et al., 2005). Besides the tests under laboratory conditions, ten participants (5 men, 5 women) performed a run of 2.4 km at a constant velocity of 10.1 km·h^−1^ under free-living conditions (Figure 1).

### Statistical analysis

Descriptive statistics (mean ± SD) summarize the characteristics of the participants, including age, weight, height and percent of body fat. All data were tested for normality with no further transformation needed. The validity of the Wearables was determined, as previously performed by other validation studies (Kooiman et al., 2015; Bai et al., 2016; An et al., 2017), by several statistical tests:

1. Systematic differences between the Wearables and the criterion measurement: mean absolute percentage error (MAPE) compared to the criterion measurement (mean difference Wearables–criterion measurement ·100· mean criterion measurement^−1^).
2. Correlation between the Wearables and the criterion measurement: Intraclass Correlation Coefficient (ICC) (two-way random, absolute agreement, single measure, 95% confidence interval) (Shrout and Fleiss, 1979), common cut-off points for validity assessment: >0.90 (excellent), 0.75–0.90 (good), 0.60–0.75 (moderate), and <0.60 (low).
3. Measure of precision: typical error (TE): TE = SD ·√1-ICC.
4. Level of agreement between the Wearables and the criterion measurement: upper and lower limits of agreement (LoA) as described by Bland-Altman.

All statistical analyses of the data were performed by using a statistics software package SPSS (version 23.0, IBM SPSS Statistics).

## Results

For the laboratory study, 20 participants were included (10 males, mean ± SD age: 26.1 ± 2.8 years; height: 182.3 ± 7.4 cm; weight: 81.1 ± 11.2 kg; body fat 11.5 ± 2.6%, and 10 females mean ± SD age: 24.2 ± 1.9 years; height: 168.2 ± 6.7 cm; weight: 60.2 ± 5.5 kg; body fat 17.9 ± 4.9%). The outdoor condition and the Withings Pulse Ox (Hip) were tested with a fewer number of participants (5 males and 5 females). Due to the high amount of lacking data, we excluded the Xaomi Miband from any data analysis.

**Figure 2:**
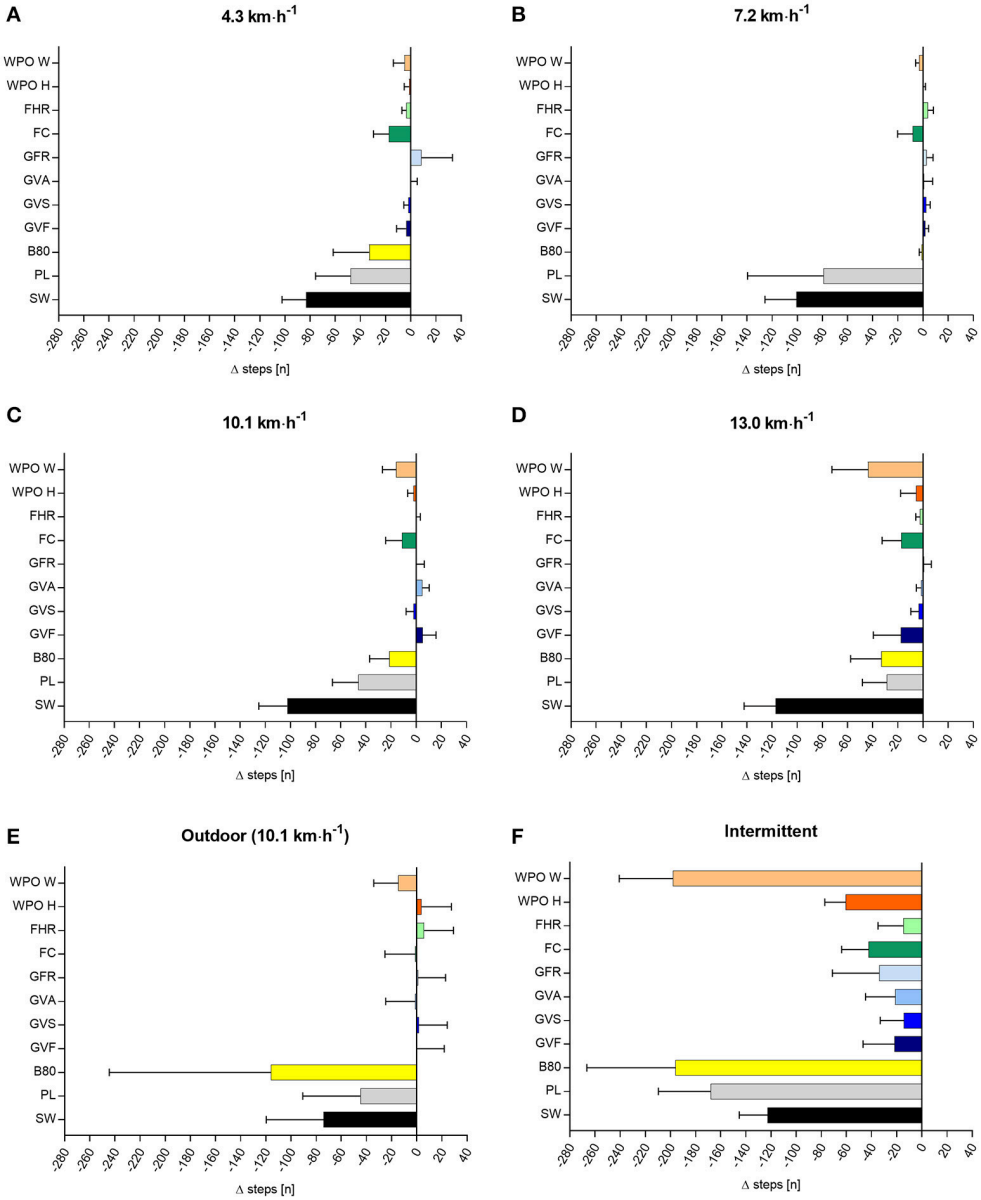
Difference in step count (n) between criterion measure and the eleven activity trackers at different running velocities **(A–F)**, data are shown as mean ± 95% CI. Mean number of steps (± SD) measured by the criterion measure: 4.3 km·h^−1^ = 538 ± 29; 7.2 km·h^−1^ = 785 ± 38; 10.1 km·h^−1^ = 822 ± 51; 13.0 km·h^−1^ = 863 ± 56; intermittent = 1,231 ± 127; outdoor = 2,456 ±145 steps. SW, Bodymedia Sensewear; PL, Polar Loop; B80, Beurer AS80; GVF, Garmin Vivofit; GVS, Garmin Vivosmart; GVA, Garmin Vivoactive; GFR, Garmin Forerunner 920XT; FC, Fitbit Charge; FHR, Fitbit Charge HR; WPO H, Withings Pulse O_x_ Hip; WPO W, Withings Pulse O_x_ Wrist.

**Figure 3:**
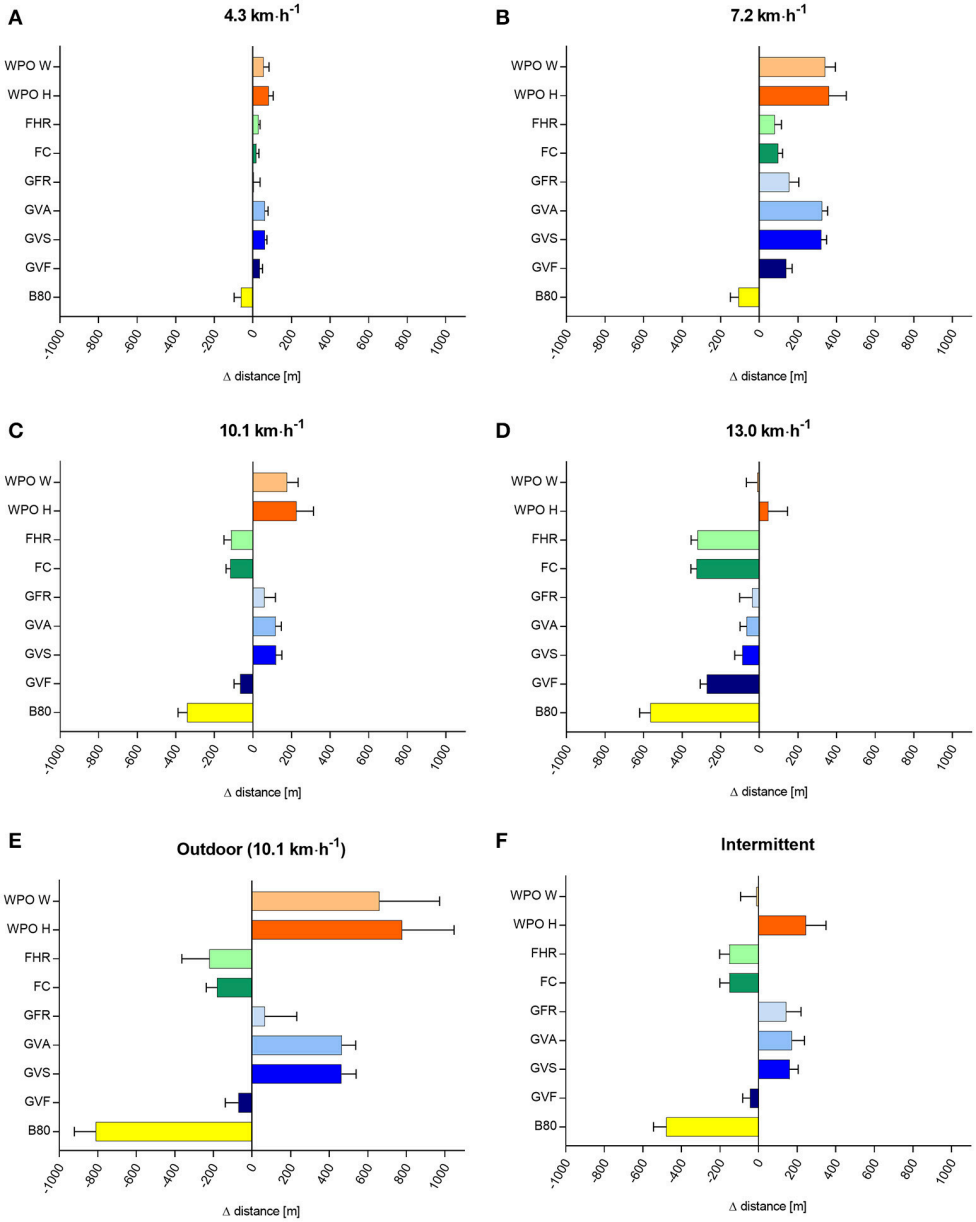
Difference in covered distance (m) between the criterion measure and the nine activity trackers at different running velocities **(A–F)**, data are shown as mean ± 95% CI. Mean covered distance (± SD) by the criterion measure were: 4.3 km·h^−1^ = 358 ± 4; 7.2 km·h^−1^ = 601 ± 6; 10.1 km·h^−1^ = 845 ± 12; 13.0 km·h^−1^ = 1,088 ± 21; intermittent = 1,139 ± 45; outdoor = 2,400 ± 0 meter. B80, Beurer AS80; GVF, Garmin Vivofit; GVS, Garmin Vivosmart; GVA, Garmin Vivoactive; GFR, Garmin Forerunner 920XT; FC, Fitbit Charge; FHR, Fitbit Charge HR; WPO H, Withings Pulse O_x_ Hip; WPO W, Withings Pulse O_x_ Wrist.

**Figure 4:**
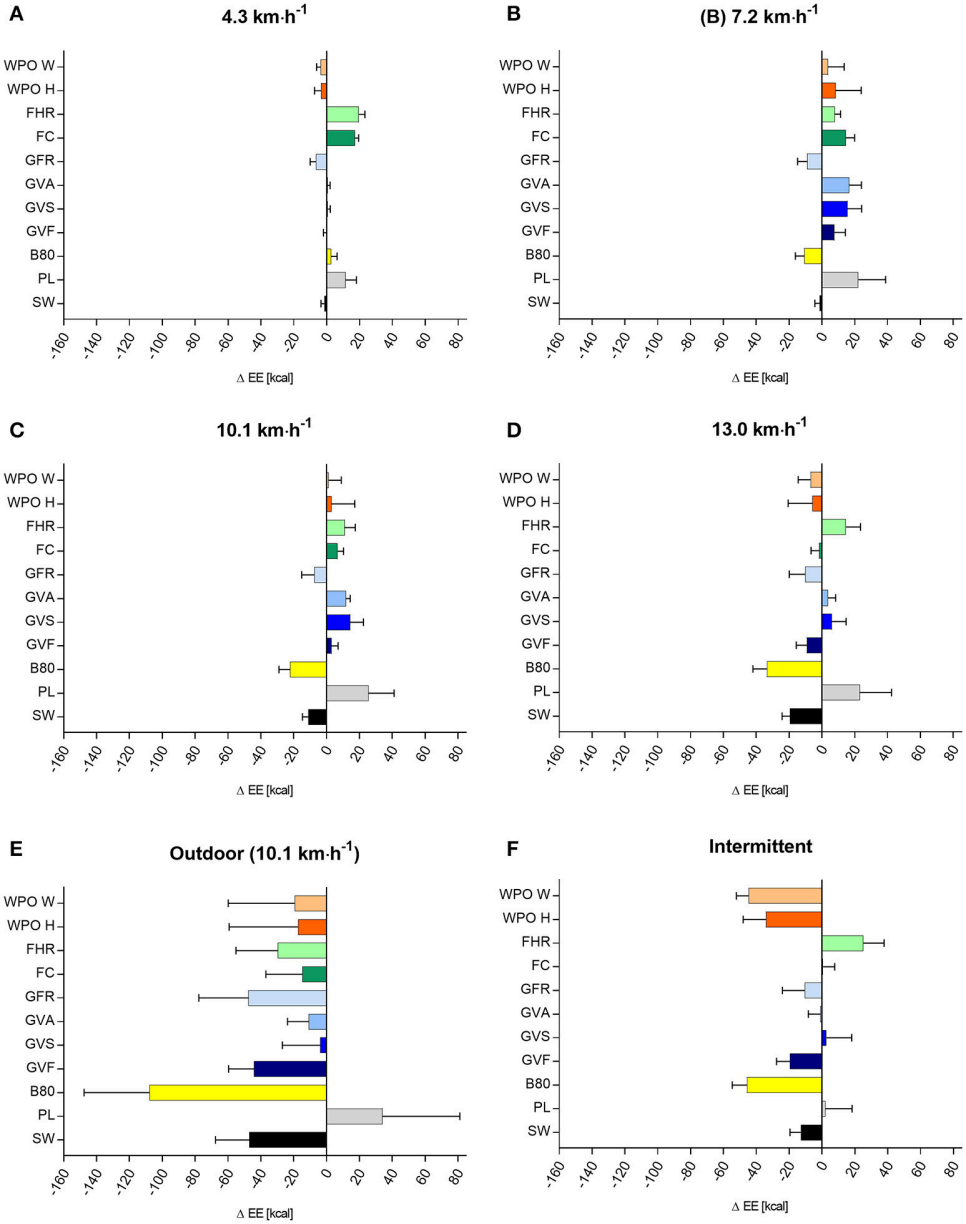
Differences in EE (kcal) between the criterion measure and the eleven activity trackers at different running verlocities **(A–F)**, data are shown as mean ± 95% CI. Mean EE (± SD) by the criterion method were: 4.3 km·h^−1^ = 24 ± 6; 7.2 km·h^−1^ = 47 ± 10; 10.1 km·h^−1^ = 61 ± 13; 13.0 km·h^−1^ = 74 ± 17; intermittent = 96 ± 18; outdoor = 210 ± 49 kcal. SW, Bodymedia Sensewear, PL, Polar Loop; B80, Beurer AS80; GVF, Garmin Vivofit; GVS, Garmin Vivosmart; GVA, Garmin Vivoactive; GFR, Garmin Forerunner 920XT; FC, Fitbit Charge; FHR, Fitbit Charge HR; WPO H, Withings Pulse O_x_ Hip; WPO W, Withings Pulse O_x_ Wrist.

The mean differences (criterion–wearable), 95% CI for step count, distance, and EE for all velocities are shown in Figures 2–4. MAPE, ICC, TE, and LoA are shown in Table 1 (step count), Table 2 (distance), Table 3 (EE).

**Table 1:** Mean absolute percentage error **(**MAPE), Intraclass Correlation Coefficient (ICC; 95%CI), typical error (TE), and upper & lower limits of agreement (LoA) for all Wearables for step count.

		**Bodymedia Sensewear**		**Polar Loop**		**Beurer AS80**		**Garmin Vivofit**		**Garmin Vivosmart**		**Garmin Vivoactive**		**Garmin Forerunner 920XT**		**Fitbit Charge**		**Fitbit Charge HR**		**Withings Pulse O**_**x**_ **Hip**		**Withings Pulse O**_**x**_ **Wrist**	
4.3 km·h^−1^	MAPE	−15.5		−8.7		−6.1		−0.7		−0.3		0.1		1.5		−3.2		−0.6		−0.2		−0.9	
	ICC (95% CI)	0.18 (−0.08–0.53)		0.06 (−0.19–0.40)		0.20 (−0.17–0.55)		0.89 (0.74–0.95)		0.97 (0.92–0.99)		0.94 (0.85–0.97)		0.72 (0.19–0.93)		0.57 (0.14–0.81)		0.96 (0.89–0.98)		0.98 (0.94–1.0)		0.82 (0.60–0.92)	
	TE	37.1		58.2		54.6		5.3		1.4		2.4		16.9		17.0		1.6		0.7		8.1	
	LoA	−3	−163	69	−165	87	−153	28	−36	14	−18	21	−20	71	−54	34	−69	12	−19	9	−12	32	−42
7.2 km·h^−1^	MAPE	−12.8		−9.6		−0.1		0.2		0.3		0.1		0.3		−1.1		0.5		−0.003		−0.4	
	ICC (95% CI)	0.18 (−0.09−0.53)		−0.27 (−0.55−0.16)		0.99 (0.98−1.0)		0.99 (0.97−1.0)		0.98 (0.96−1.0)		0.94 (0.85−0.98)		0.98 (0.94−1.0)		0.83 (0.62−0.93)		0.97 (0.91−0.99)		0.59 (0.03−0.88)		0.99 (0.99−1.0)	
	TE	48.5		110.2		0.4		0.6		0.9		3.6		1.0		10.6		1.6		1.6		0.6	
	LoA	4	−210	174	−332	7	−9	13	−9	16	−11	30	−28	17	−11	42	−58	22	−14	5	−5	8	−15
10.1 km·h^−1^	MAPE	−12.5		−5.4		−2.5		0.7		−0.2		0.6		0.04		−1.3		0.01		−0.2		−1.9	
	ICC (95% CI)	0.27 (−0.08−0.65)		0.39 (−0.08−0.72)		0.72 (0.34−0.89)		0.91 (0.78−0.96)		0.97 (0.93−0.99)		0.97 (0.93−0.99)		0.99 (0.96−1.0)		0.85 (0.66−0.94)		0.99 (0.98−1.0)		0.99 (0.98−1.0)		0.83 (0.51−0.94)	
	TE	41.2		34.5		17.9		6.8		2.2		2.0		0.9		10.8		0.7		0.7		9.6	
	LoA	−8	−197	41	−133	45	−88	50	−39	23	−27	27	−17	17	−17	43	−66	14	−13	11	−15	30	−62
13.0 km·h^−1^	MAPE	−13.5		−3.3		−3.7		−2.0		−0.4		−0.2		0.1		−1.9		−0.3		−0.6		−4.8	
	ICC (95% CI)	0.23 (−0.08−0.60)		0.69 (0.26−0.88)		0.49 (0.07−0.76)		0.73 (0.43−0.88)		0.97 (0.93−0.99)		0.99 (0.98−1.00)		0.99 (0.97−1.00)		0.78 (0.49−0.91)		0.99 (0.98−1.00)		0.96 (0.86−0.99)		0.25 (−0.11−0.59)	
	TE	47.2		23.0		37.3		24.4		2.3		0.8		0.8		15.2		0.7		3.2		53.0	
	LoA	−12	−223	52	−110	69	−135	75	−109	23	−30	15	−17	17	−15	46	−81	11	−16	25	−37	76	−164
Inter−mittent	MAPE	−9.9		−13.3		−16.2		−1.4		−1.1		−1.5		−2.7		−3.3		−1.1		−4.7		−16.0	
	ICC (95% CI)	0.62 (−0.06−0.90)		0.31 (0.09−0.70)		0.37 (−0.11−0.72)		0.88 (0.71−0.95)		0.95 (0.87−0.98)		0.89 (0.74−0.96)		0.93 (0.72−0.98)		0.88 (0.43−0.96)		0.93 (0.84−0.97)		0.91 (−0.02−0.99)		0.34 (−0.08−0.73)	
	TE	29.7		73.9		119.5		17.9		9.0		16.8		13.8		16.0		11.5		7.0		74.0	
	LoA	−28	−127	7	−342	99	−491	80	−123	64	−93	78	−121	69	−136	48	−133	71	−100	−15	−106	−20	−377
Outdoor	MAPE	−3.0		−1.9		−4.7		−0.02		−0.04		−0.1		0.03		−0.1		0.2		0.1		−0.6	
	ICC (95% CI)	0.82 (0.07−0.96)		0.83 (0.32−0.96)		0.50 (−0.06−0.84)		0.98 (0.93−1.0)		0.98 (0.92−1.0)		0.98 (0.92−1.0)		0.98 (0.93−1.0)		0.98 (0.91−0.99)		0.98 (0.92−0.99)		0.98 (0.92−1.0)		0.98 (0.91−1.0)	
	TE	27.1		24.5		127.2		4.3		4.5		4.6		4.3		4.8		4.6		4.3		3.8	
	LoA	51	−199	72	−161	237	−468	60	−60	63	−60	63	−65	61	−58	65	−67	70	−58	64	−56	38	−68

**Table 2:** Mean absolute percentage error **(**MAPE), Intraclass Correlation Coefficient (ICC; 95%CI), typical error (TE), and upper & lower limits of agreement (LoA) for all Wearables for covered distance.

		**Beurer AS80**		**Garmin Vivofit**		**Garmin Vivosmart**		**Garmin Vivoactive**		**Garmin Forerunner 920XT**		**Fitbit Charge**		**Fitbit Charge HR**		**Withings Pulse O**_**x**_ **Hip**		**Withings Pulse O**_**x**_ **Wrist**	
4.3 km·h^−1^	MAPE	−17.6		9.8		17.7		17.4		1.3		5.3		8.0		19.0		17.8	
	ICC (95% CI)	0.01 (−0.22−0.32)		0.02 (−0.30−0.27)		0.01 (−0.03−0.08)		0.004 (−0.08−0.16)		0.003 (−0.46−0.45)		−0.003 (−0.27−0.35)		0.02 (−0.08−0.22)		0.01 (−0.05−0.20)		−0.01 (−0.19−0.55)	
	TE	73.3		30.3		19.6		34.5		69.2		27.1		17.9		32.1		57.8	
	LoA	82	−207	95	−25	102	25	130	−6	141	−131	72	−34	64	−7	145	19	169	−57
7.2 km·h^−1^	MAPE	−18.1		23.3.		53.5		51.4		26.0		16.0		13.1		57.6		58.3	
	ICC (95% CI)	−0.02 (−0.15−0.22)		−0.01 (−0.06−0.11)		−0.002 (−0.01−0.02)		−0.002 (−0.01−0.02)		−0.003 (−0.11−0.19)		−0.01 (−0.09−0.14)		−0.02 (−0.19−0.25)		−0.003 (−0.05−0.14)		−0.003 (−0.03−0.6)	
	TE	90.2		64.4		59.0		59.3		102.9		52.5		76.5		126.5		111.6	
	LoA	66	−284	266	14	436	206	441	208	357	−45	198	−6	227	−70	608	126	560	124
10.1 km·h^−1^	MAPE	−40.3		−7.8		14.2		13.9		7.0		−13.9		−13.4		24.3		22.3	
	ICC (95% CI)	−0.003 (−0.03−0.05)		0.05 (−0.21−0.35)		−0.01 (−0.08−0.14)		−0.03 (−0.10−0.13)		−0.05 (−0.38−0.35)		−0.01 (−0.05−0.09)		−0.01 (−0.12−0.19)		−0.01 (−0.10−0.25)		−0.02 (−0.13−0.18)	
	TE	101.6		67.1		64.6		65.1		125.9		48.4		78.7		123.8		122.8	
	LoA	−142	−540	69	−201	244	−6	243	−9	300	−182	−23	−212	40	−267	467	−16	415	−61
13.0 km·h^−1^	MAPE	−51.9		−25.0		−8.1		−6.1		−3.3		−29.9		−29.5		1.0		0.7	
	ICC (95% CI)	−0.005 (−0.02−0.03)		0.007 (−0.02−0.07)		−0.10 (−0.28−0.20)		−0.04 (−0.23−0.26)		−0.15 (−0.54−0.30)		−0.002 (−0.01−0.02)		−0.001 (−0.02−0.03)		0.04 (−0.57−0.62)		−0.11 (−0.55−0.36)	
	TE	119.7		74.1		81.8		72.6		149.9		61.7		71.3		137.3		127.0	
	LoA	−331	−799	−127	−418	70	−249	73	−206	237	−311	−205	−447	−181	−461	321	−229	225	−247
Intermittent	MAPE	−42.3		−3.7		14.2		15.5		12.4		−13.3		−13.2		−0.9		10.2	
	ICC (95% CI)	0.05 (−0.03−0.22)		0.28 (−0.11−0.62)		0.08 (−0.08−0.34)		−0.07 (−0.21−0.19)		0.17 (−0.13−0.50)		0.11 (−0.10−0.41)		0.07 (−0.10−0.33)		0.09 (−0.10−0.46)		0.10 (−0.37−0.52)	
	TE	139.2		67.1		89.5		142.4		149.5		99.3		104.6		139.6		169.1	
	LoA	−198	−758	110	−199	344	−22	443	−97	465	−178	55	−358	61	−364	532	−42	339	−360
Outdoor	MAPE	−33.8		−3.2		17.7		15.5		9.7		−10.1		−9.7		16.7		27.3	
	ICC (95% CI)	0.000 (−0.01−0.05)		0.000 (−0.34−0.50)		0.000 (−0.02−0.07)		0.000 (−0.02−0.06)		0.000 (−0.62−0.61)		0.000 (−0.07−0.20)		0.000 (−0.22−0.42)		0.000 (−0.08−0.27)		0.000 (−0.13−0.32)	
	TE	156.2		93.9		104.6		99.8		231.5		79.2		200.2		375.9		436.0	
	LoA	−504	−1116	112	−256	669	259	661	270	520	−388	−26	−336	171	−613	1515	42	1515	−194

**Table 3:** Mean absolute percentage error **(**MAPE), Intraclass Correlation Coefficient (ICC; 95%CI), typical error (TE), and upper & lower limits of agreement (LoA) for all Wearables for energy expenditure.

		**Bodymedia Sensewear**		**Polar Loop**		**Beurer AS80**		**Garmin Vivofit**		**Garmin Vivosmart**		**Garmin Vivoactive**		**Garmin Forerunner 920XT**		**Fitbit Charge**		**Fitbit Charge HR**		**Withings Pulse O**_**x**_ **Hip**		**Withings Pulse O**_**x**_ **Wrist**	
4.3 km·h^−1^	MAPE	−4.3		56.4		17.0		1.3		2.9		4.0		−26.6		75.0		83.3		−9.9		−11.0	
	ICC (95% CI)	0.61 (0.25−0.82)		−0.11 (−0.26−0.31)		−0.17 (−0.54−0.27)		0.73 (0.42−0.88)		0.91 (0.78−0.96)		0.85 (0.66−0.94)		0.35 (−0.08−0.68)		0.15 (−0.04−0.50)		0.17 (−0.06−0.52)		0.44 (0.11−0.81)		0.28 (−0.10−0.62)	
	TE	2.6		9.6		8.5		2.2		0.9		1.1		6.0		4.6		7.2		4.3		4.4	
	LoA	6.6	−9.7	29.5	−6.4	18.1	−12.9	8.1	−8.2	6.5	−5.0	6.4	−5.0	8.0	−21.1	26.8	7.4	35.0	4.2	7.8	−14.6	6.6	−13.9
7.2 km·h^−1^	MAPE	−1.4		53.8		−18.9		18.7		35.8		36.8		−16.7		33.6		18.2		18.6		16.9	
	ICC (95% CI)	0.77 (0.50−0.90)		0.02 (−0.25−0.48)		−0.09 (−0.29−0.22)		0.35 (−0.05−0.67)		0.21 (−0.12−0.55)		0.28 (−0.12−0.55)		0.25 (−0.11−0.58)		0.29 (−0.11−0.64)		0.58 (−0.02−0.84)		−0.15 (−0.68−0.52)		−0.15 (−0.56−0.31)	
	TE	2.9		22.9		12.1		11.5		16.3		13.8		10.8		9.8		5.0		21.8		22.2	
	LoA	10.5	−13.4	67.6	−23.1	12.0	−33.5	35.5	−20.2	51.6	−20.4	48.3	−15.3	15.5	−33.4	37.2	−8.2	22.9	−7.3	48.1	−31.5	44.5	−36.8
10.1 km·h^−1^	MAPE	−17.2		51.2		−33.0		6.5		24.0		20.2		−9.3		13.5		20.4		9.4		5.4	
	ICC (95% CI)	0.52 (−0.10−0.83)		−0.09 (−0.26−0.33)		−0.04 (−0.15−0.16)		0.76 (0.49−0.90)		0.46 (−0.01−0.76)		0.69 (−0.07−0.92)		0.25 (−0.15−0.60)		0.68 (0.13−0.88)		0.43 (−0.01−0.73)		0.27 (−0.51−0.78)		0.20 (−0.28−0.59)	
	TE	5.1		22.8		14.9		4.3		12.6		3.1		14.3		4.3		10.6		15.7		15.2	
	LoA	3.1	−25.7	68.3	−17.3	6.5	−50.7	20.0	−14.1	48.0	−19.1	22.8	0.8	25.1	−39.8	21.7	−8.2	38.4	−16.4	39.1	−33.0	34.3	−32.1
13.0 km·h^−1^	MAPE	−25.3		41.2		−42.7		−11.1		9.0		6.2		−11.1		−0.1		22.2		−8.4		−5.3	
	ICC (95% CI)	0.39 (−0.1−0.76)		−0.25 (−0.45−0.33)		−0.02 (−0.09−0.13)		0.56 (0.12−0.81)		0.59 (0.23−0.81)		0.82 (0.59−0.92)		0.28 (−0.11−0.62)		0.73 (0.43−0.88)		0.43 (−0.01−0.73)		0.54 (−0.12−0.87)		0.46 (0.05−0.74)	
	TE	8.2		30.4		18.0		9.0		11.8		4.2		17.6		5.5		14.2		13.1		11.4	
	LoA	1.1	−39.8	76.3	−30.3	1.4	−68.6	17.3	−35.9	42.2	−30.0	23.0	−15.6	30.5	−50.8	18.8	−22.3	51.5	−22.1	32.0	−43.5	23.4	−37.3
Intermittent	MAPE	−12.4		5.6		−45.9		−21.3		2.0		−1.3		−9.2		2.4		25.5		−48.8		−38.9	
	ICC (95% CI)	0.49 (−0.02−0.78)		−0.30 (−0.89−0.43)		0.000 (−0.05−0.10)		0.54 (−0.10−0.84)		0.43 (−0.02−0.73)		0.74 (0.44−0.89)		0.22 (−0.19−0.59)		0.58 (0.19−0.81)		0.43 (−0.05−0.74)		0.01 (−0.10−0.29)		0.11 (−0.05−0.40)	
	TE	10.0		25.9		18.9		11.8		24.8		7.9		25.8		9.7		20.4		19.2		14.8	
	LoA	14.6	−40.5	46.5	−42.5	−8.8	−82.8	11.4	−53.5	67.0	−61.6	29.4	−31.5	46.9	−67.8	30.0	−28.5	78.1	−27.7	3.7	−72.1	−14.0	−75.5
Outdoor	MAPE	−20.8		22.1		−48.4		−20.2		−1.5		−4.5		−21.2		−4.5		−12.0		−5.5		−4.5	
	ICC (95% CI)	0.43 (−0.11−0.82)		−0.18 (−0.56−0.48)		−0.04 (−0.12−0.22)		0.56 (−0.09−0.89)		0.82 (0.43−0.95)		0.91 (0.64−0.98)		0.34 (−0.14−0.77)		0.64 (0.11−0.89)		0.53 (−0.05−0.85)		0.21 (−0.44−0.72)		0.22 (−0.41−0.72)	
	TE	21.8		71.4		56.8		14.3		13.6		5.4		31.9		18.6		24.4		52.0		50.0	
	LoA	9.7	−103.6	163.0	−94.8	1.3	−216.9	−1.9	−86.6	59.0	−66.8	24.3	−46.2	29.3	−124.5	46.2	−75.6	40.2	−99.5	97.3	−132.2	91.7	−130.4

### Step count

The mean step count (± SD) measured by the criterion measure was: 538 ± 29 (4.3 km·h^−1^); 785 ± 38 (7.2 km·h^−1^); 822 ± 51 (10.1 km·h^−1^); 863 ± 56 (13.0 km·h^−1^); 1,231 ± 127 (intermittent); 2,456 ± 145 (outdoor) steps. Bodymedia Sensewear, Polar Loop, and Beurer AS80 showed a substantial MAPE up to 16%, a low to moderate ICC, a large TE (up to 100 steps), and the broadest LoA. The other Wearables showed a small MAPE (<2%) for all test conditions as well as a good to excellent ICC. Garmin Vivosmart, Garmin Vivoactive, Fitbit Charge HR, Withings Pulse Ox Hip showed a small TE, and the narrowest LoA.

### Covered distance

The mean covered distance (± SD) by the criterion measure was: 358 ± 4 (4.3 km·h^−1^); 601 ± 6 (7.2 km·h^−1^); 845 ± 12 (10.1 km·h^−1^); 1,088 ± 21 (13.0 km·h^−1^); 1,139 ± 45 (intermittent); 2,400 ± 0 (outdoor) m. Beurer AS80 showed a high MAPE (17.6 up to 51.9%) for all test conditions. Garmin Vivofit, Vivosmart, Vivoactive, Forerunner, Fibit Charge, Charge HR and Withings showed a moderate MAPE (1.3–29.9%) for all test conditions expect 7.2 km·h^−1^. The ICC for all Wearables was very low (<0.1). Garmin Vivosmart, Garmin Vivoactive, Fitbit Charge, and Fitbit Charge HR showed a small TE, and the narrowest LoA.

### Energy expenditure

The mean EE (± SD) by the criterion measure were: 24 ± 6 (4.3 km·h^−1^); 47 ± 10 (7.2 km·h^−1^); 61 ± 13 (10.1 km·h^−1^); 74 ± 17 (13.0 km·h^−1^); 96 ± 18 (intermittent); 210 ± 49 (outdoor) kcal.

Bodymedia Sensewear, Polar Loop, Beurer AS80 showed a high MAPE up to 56% for all test conditions. The Garmin, Fitbit and Withings Wearables showed a small to moderate MAPE (1.3–21.2 %) for 10.1 km·h^−1^, 13.0 km·h^−1^, and the Outdoor condition. Garmin Vivofit, Vivosmart, Vivoactive, Fitbit Charge and Charge HR showed a moderate to good ICC, whereas Bodymedia Sensewear, Polar Loop, Beurer AS80, Garmin Forerunner 920XT and Withings Pulse Ox showed a low ICC. Bodymedia Sensewear, Garmin Vivofit, Garmin Vivoactive, Fitbit Charge showed a small TE, and the narrowest LoA.

## Discussion

The aim of the present study was to investigate the criterion-validity of eleven Wearables for step count, covered distance and EE over a large spectrum of constant and intermittent velocities reflecting sports conditions. The results indicate that most Wearables, except Beurer AS80, Polar Loop, Bodymedia Sensewear provide an acceptable level of validity concerning step count for all constant velocities, the intermittent protocol as well as for the outdoor condition. The parameters covered distance and EE, however, exhibited a low validity for any of the conditions for most of the Wearables. The Xaomi Miband did lack a high amount of data and we, therefore, want to discourage using this Wearable to monitor step count, distance, and EE in sports conditions.

### Step count

In line with the present study, other laboratory-based studies also showed generally high correlations for step count between the criterion measure and Wearables (Takacs et al., 2014; Diaz et al., 2015; Evenson et al., 2015). Tudor-Locke et al. (2006) stated that Wearables generally should not exceed a MAPE of 1% compared to the criterion measure during walking on a treadmill at a speed of 4.8 km·h^−1^ in order to be considered accurate. Garmin Vivosmart, Garmin Vivoactive, Garmin Forerunner 920 XT, Fitbit Charge HR, and Withings Pulse O_x_ (Hip) had a MAPE <1% over all test conditions. Fitbit Charge and Garmin Vivofit had a slightly higher MAPE of <3%, still representing good results. Bodymedia Sensewear, Polar Loop, and Beurer AS80 had MAPE between 3.7 and 15.5%, whereby all devices underestimated the number of steps taken. When errors were higher, the direction tended to be an under-estimation of step count by the tracker compared to the criterion. This may be particularly problematic at slow walking speeds (Evenson et al., 2015). Garmin Vivosmart, Garmin Vivoactive, Fitbit Charge HR, and Withings Pulse Ox indicated the narrowest LoA (less than 50 steps for the constant velocities). This can be considered as a relatively small range. The range between the upper and lower LoA of Bodymedia Sensewear, Polar Loop, and Beurer AS80 (up to 200 steps) are considered to be too large to be used interchangeably with the criterion measure. In a sport specific condition like a marathon run with an average velocity of 10.1 km·h^−1^ an average step count of 60.000 steps represents an error of +60 steps for Fitbit Charge HR or −7.500 steps for Bodymedia Sensewear.

For the intermittent velocities, which are typical for most sport disciplines, the discrepancy was high, revealing an underestimation for all Wearables between −14 ± 40 steps (Garmin Vivosmart) up to −198 ± 91 (Withings Pulse O_x_ Wrist). For intermittent sports, like a 90 min competitive soccer game, players will cover on average about 13.000 steps, which represents a small error of −143 steps for Fitbit Charge HR/Garmin Vivosmart up to a high underestimation of 2.106 steps for Beurer AS80.

The outdoor condition, which resembled the same velocity as the third speed on the treadmill (10.1 km·h^−1^), showed similar results as the laboratory testing using constant velocities.

In summary, the step count for most of the Wearables, except Bodymedia Sensewear, Polar Loop, and Beurer AS80 showed to be valid. However, generally, there is a tendency to underestimate the number of steps. One might speculate, that a reduced arm movement while walking/running leads to an underestimation of the step count. Furthermore, it might be a problem of the adjustment of the sensitivity of the accelerometers and different algorithms. The manufacturers have the problem, that wearables should not count every single arm movement during daily life as a step. Therefore, the acceleration needs to exceed a certain threshold to be processed by the algorithm and to be counted as a step.

### Covered distance

The measurement of covered distance showed no consistent discrepancy over the different velocities between the Wearables and the criterion measure. The Wearables mainly showed an overestimation of distance for constant slower velocities (4.3 and 7.2 km·h^−1^) and an underestimation of distance for higher velocities (13.0 km·h^−1^). This is in line with the study of Takacs et al. (2014), showing an overestimation for slower speeds (3.2–4.7 km·h^−1^) and an underestimation for faster speeds (6.4 km·h^−1^). In elite sport fast running velocities often occur, and consequently, the covered distance will be underestimated in these instances with the presented Wearables. The highest MAPE (−18.1 to 58.3%) of all Wearables was reached at the velocity of 7.2 km·h^−1^, whereas the lower velocity of walking (4.3 km·h^−1^) showed a better MAPE (1.3 to 19%). The ICC ranged from 0.0 to 0.2 for all tested conditions, indicating poor agreement with the criterion measure. This is line with the study of Takacs et al. (2014), showing small ICC between 0.0 and 0.05. Although Garmin Vivosmart, Garmin Vivoactive, Fitbit Charge, and Fitbit Charge HR showed the narrowest LoA, the range is still insufficiently high. In sport specific situations, like a marathon run at 10.1 km·h^−1^, covered distance will be overestimated by ~2.94 km with Garmin Forerunner 920XT, or underestimated by ~16.9 km with Beurer AS80.

In the intermittent protocol, the covered distance derived from Wearables show a high discrepancy compared to the criterion measure, with some Wearables overestimating (Withings Pulse Ox Hip, Garmin Forerunner 920XT, Garmin Vivoactive, Garmin Vivosmart), others underestimating this parameter (Fitbit Charge HR, Fitbit Charge, Garmin Vivofit, Beurer AS80). For intermittent sports, like a 90 min soccer game (mean distance 12 km), the covered distance will be underestimated by ~1.080 m using Withings Pulse Ox hip up to ~5.076 m using Beurer AS80 based on our findings.

The outdoor condition (10.1 km·h^−1^) showed similar high MAPE compared to the laboratory condition with the same Wearables overestimating (Withings Pulse O_x_ Wrist and Hip, Garmin Forerunner 920XT, Garmin Vivoactive, Garmin Vivosmart) or underestimating (Fitbit Charge HR, Fitbit Charge, Garmin Vivofit, Beurer AS80) the covered distance.

In summary, for monitoring the covered distance, no Wearable could achieve good validity for all laboratory-based constant and intermittent velocities as well as in the outdoor condition. We acknowledge that the covered distance can be assessed by other Wearables employing for example receivers for Global Navigation Satellite Systems such as Global Positioning Systems (Cummins et al., 2013) and it seems that this technology is superior to accelerometry to derive the covered distance in sports conditions.

### Energy expenditure

The measurement of EE showed no consistent discrepancy over the different velocities between the Wearables and the criterion measure. The Wearables mainly showed an overestimation of EE for constant slower velocities (4.3; 7.2; 10.1 km·h^−1^) and an underestimation of EE for higher velocities (13.0 km·h^−1^). Overall, Bodymedia Sensewear, Polar Loop, Beurer AS80 showed a low validity for all test conditions. The Garmin, Fitbit and Withings Wearables showed a better validity with small to moderate MAPE (1.3–21.2%) for the faster velocities (10.1 km·h^−1^, 13.0 km·h^−1^). The results are in line with a review of Evenson et al. (2015) showing a low validity for EE in 10 adult studies. Although Bodymedia Sensewear, Garmin Vivofit, Garmin Vivoactive, and Fitbit Charge showed the narrowest LoA, the range is still insufficiently high. The ICC ranged from moderate to substantial agreement, while larger bias show the tendency to underestimate EE. Extrapolated to a marathon run (~3,000 kcal), this equates to an error of ~86 kcal overestimation for Withings Pulse Ox Wrist up to ~820 kcal for Polar Loop for a runner of 70 kg with a finishing time of 4:13 h (McArdle et al., 2000).

Fitbit Charge, Garmin Vivoactive, Garmin Vivosmart, and Polar Loop showed relative small MAPE (<5.6%) for the intermittent protocol, whereas the other devices mainly underestimate the EE (Withings Pulse O_x_ (Wrist or Hip), Garmin Forerunner 920XT, Garmin Vivofit, Beurer AS80, Bodymedia Sensewear). For intermittent sports, like a 90 min soccer game (mean EE ~1300 kcal), EE will be underestimated by ~17 kcal using Garmin Vivoactive up to ~630 kcal using Withings Pulse O_x_ hip.

The outdoor condition showed a completely contrary pattern compared to the laboratory condition (10.1 km·h^−1^). While all devices underestimate the EE in the outdoor condition, most of the devices overestimate EE in the comparable laboratory condition. This is surprising, but may be an issue of reliability, an aspect we intentionally did not target in our study. To clarify this, we want to encourage researchers in conducting reliability studies on the presented Wearables. In summary, the presented Wearables should be used very cautiously to assess EE.

## Limitations

Generally, we have to acknowledge some limitations of the present study. First, there might be some limitations arising from calculating EE via indirect calorimetry using the device Metamax 3B (Lighton, 2008). Even though the experiments were conducted within 2 weeks of time, which might limit the degradation of the oxygen sensor, previous studies showed, that the Metamax 3B produces acceptably stable and reliable results, but is not adequately valid during moderate and vigorous exercise without some further correction of VO_2_ and VCO_2_ (Macfarlane and Wong, 2012). As in every validation study, we cannot be entirely sure if some error arises from the criterion-measure and encourage to see the results of this study in light of these limitations.

Second, the velocities on the treadmill were not randomized, as we expected that higher velocities would influence slower velocities more than the other way round. Therefore, we decided not to randomize the velocities, but to gradually increase the velocity. Additionally, during the 5 min rest periods, spirometric and heart rate values decreased to resting levels. Anyhow, we cannot completely discard a cardiovascular drift.

Third, in comparison to several previous validation studies (Kooiman et al., 2015; Bai et al., 2016; An et al., 2017), we investigated a similar number of subjects. However, the relatively small sample size might limit the statistical power of the present results. There are several statistical approaches for validation studies. However, possibly no statistical approach will remain uncriticised and every approach has its advantages and drawbacks. According to previously published validation studies (Kooiman et al., 2015; Bai et al., 2016; An et al., 2017), we used the statistical approach from this studies.

## Conclusion

In our study, most Wearables provide an acceptable level of validity for step counts at different constant and intermittent running velocities reflecting sports conditions. The most valid Wearables, represented by the smallest MAPE, to monitor step count were Garmin Vivosmart, Garmin Vivoactive, Garmin Forerunner 920XT, Fitbit Charge, Fitbit Charge HR and Withings Pulse Ox (Hip). Yet, the covered distance, as well as the EE, could not be assessed validly with the investigated Wearables. Especially in sport specific conditions, like a marathon run or a 90 min soccer game, covered distance and EE showed high errors for nearly all Wearables. Consequently, covered distance and EE should not be monitored with the presented Wearables.

## Author contributions

All authors listed have made a substantial, direct and intellectual contribution to the work, and approved it for publication.

### Conflict of interest statement

The authors declare that the research was conducted in the absence of any commercial or financial relationships that could be construed as a potential conflict of interest.
